# Supplementary material for: Epidemiology and clinical characteristics of viral infections in hospitalized children and adolescents with cancer in Lebanon
Source: PLoS One. 2020 Sep 22;15(9):e0239258. doi: 10.1371/journal.pone.0239258 (PMC7508634; doi:10.1371/journal.pone.0239258)
Supplement: Attachment — Submitted filename: Response to Reviewers.docx [file pone.0239258.s002.docx]

PONE-D-19-32741R1
Epidemiology and clinical characteristics of viral infections in hospitalized children and adolescents with cancer in Lebanon
PLOS ONE

Eric HY Lau, Ph.D.	August 16, 2020
Academic Editor
PLOS ONE

Dear Dr. Lau,

We would like to thank you and reviewer #2 for taking the time to review our paper and provide us with additional constructive feedback. Thank you for finding that our manuscript has merit, and we hope that with the enclosed edits you will find it publishable in PLOS One. Below is a point-by-point response to the comments.

**Editor’s Comment:**

**“Tables 4, 6, please remove Fisher’s Exact test in the footnote. You may also indicate why the test was not carried out.”**

We have removed the Fisher’s Exact test in the footnote as suggested.

We thank **Reviewer #2** for her thoughtful and helpful comments. Please find below the comments and their respective responses:

1. *In the conclusion, the authors state that viral infections contributed significantly to morbidity and mortality. It is difficult to conclude that this was the case without data on the same variable in a comparison group (those with no viral infections). It is indeed possible that the morbidity and mortality was comparable in both groups.
   In addition, there is no definite evidence from the data that the cause of death in these patients was the viral infections. Cancer patients commonly have multimorbidity, and potential multiple causes of death. It might be better to make these statements in a probabilistic manner*.

Response: We agree with the reviewer and have changed the wording of the opening sentence of the abstract conclusion to indicate that viral infections “may” have contributed to morbidity and mortality.

2*. The terms patients and subjects are used interchangeably. Ethically, it is better to refer to them as participants. Once an individual is on a study, even retrospectively, they are referred to as participants, and this term needs to be used wherever appropriate*.

Response: We thank the reviewer for this suggestion. We replaced all "subjects" and most “patients” with "participants" with the exception of text referring to patients prior to them becoming participants, which were referred to as "patients" (i.e. while describing methods and selection process)

3. *Line 619-626 has scientific facts, but it is not clear how they relate to the current study. Were there any participants who had the complications mentioned? If so, state that and discuss that result*.

Response: We thank the reviewer for this comment. The two references relating to secondary bacterial complications of influenza are not immediately relevant to our findings, so we decided to eliminate them. We have modified the language of these lines to read as follows: “On the other hand, the burden of RSV in the pediatric population extends beyond infancy where it may cause bronchiolitis, bronchospasm, pneumonia and acute respiratory failure especially in children with co-morbidities with a mortality rate ranging between 1.6% in older children and 2.3% in neonates (35). Although we encountered one mortality each from RSV and influenza in our participants, it was difficult to attribute these mortalities to the viral infections because of the presence of other co-morbidities”.

4. *The results showed that it was not possible to get a record of the participants’ vaccination status. I propose that make note of this and discuss the wide implications in terms of general child health but also in the context of cancer patients, and probably a recommendation on improvements on data capture on vaccination.*

Response: We thank the reviewer for this suggestion. We have emphasized this in the “Limitations” and included the following statement in the “Conclusion”: “In addition, the difficulty encountered in obtaining vaccination status from medical records for vaccine-preventable viral infections in the study such as VZV, rotavirus, and influenza highlights the importance of capturing this information in the record and engaging the primary care provider in the care of these patients”.

5. *In the conclusion, the authors make a note of the importance of rational antibiotic use which can be guided by a high index of suspicion of viral infections by the clinicians. However, they backtrack on this important issue by suggesting that this can happen after failure of broad-spectrum and this defeats the argument on rational antibiotic use.*

Response: We thank the reviewer for pointing this out and we agree that this defeats the argument. We have changed the wording of the relevant sentences in the “Conclusion” to the following: “With the rising rates of antimicrobial resistance and the resulting efforts at antimicrobial stewardship, the identification of viral causes of infection can help limit the unnecessary and prolonged use of antibiotics in the pediatric cancer population especially for those presenting with fever and neutropenia. Testing for infection with most of the viruses reported in this study can be easily performed upon presentation. Thus, we recommend that physicians maintain a high degree of suspicion for viral infection and consider early testing in immunocompromised patients who present with compatible signs and symptoms in order to spare the use of antibiotics”.

6. *Generally, the conclusion section seems more focused on recommendations (which is fine) but I miss specific concluding statements related to the study objective (s) and results*.

Response: We thank the reviewer for pointing this omission. We have restructured the “Conclusion” to highlight the findings from study in the beginning and to provide the recommendations afterwards.

For the authors,

Ghassan Dbaibo, M.D., FAAP
Professor and Vice-Chair for Research and Faculty Development
Department of Pediatrics and Adolescent Medicine
Head, Division of Pediatric Infectious Diseases
Director, Center for Infectious Diseases Research
Co-Director, Primary Immunodeficiency Diseases Program
Professor, Department of Biochemistry and Molecular Genetics
American University of Beirut
PO Box 11-0236
Riad El-Solh
Beirut, Lebanon

Tel: +961-1374374 
ext: 5540 Hospital Office
ext: 5752 Clinical Research Office
ext: 4819 Biochemistry
Fax: +961-1-370781

[gdbaibo@aub.edu.lb](mailto:gdbaibo@aub.edu.lb)
